# Elevated levels of C-reactive protein and pig major acute phase protein in lame gestating sows

**DOI:** 10.3389/fvets.2025.1505132

**Published:** 2025-02-19

**Authors:** Nadia Jakobsen, Inge Larsen, Nicolai R. Weber, Peter M. H. Heegaard, Ken S. Pedersen

**Affiliations:** ^1^Department of Veterinary and Animal Sciences, Faculty of Health and Medical Sciences, University of Copenhagen, Frederiksberg, Denmark; ^2^Danish Agriculture & Food Council F.m.b.A., Copenhagen, Denmark; ^3^Department of Health Technology, Technical University of Denmark, Kgs. Lyngby, Denmark; ^4^Ø-Vet A/S, Næstved, Denmark

**Keywords:** serum amyloid A, C-reactive protein, pig major acute phase protein, haptoglobin, lameness, diagnostics, sows

## Abstract

**Introduction:**

Lameness is a common clinical issue in gestating sows and a leading cause for early culling and antibiotic treatment. Diagnostic tools are limited, but acute acute phase proteins (APPs) could offer a fast and minimally invasive method for optimizing treatment. The aim of this study was to investigate whether APP level measurements can be used as a diagnostic tool, and to identify which major acute phase proteins are elevated in lame gestating sows.

**Methods:**

To determine this, blood samples were collected from the jugular vein of 50 lame and 50 clinically healthy gestating sows from 12 conventional herds. The samples were analyzed for C-reactive protein (CRP), haptoglobin, serum amyloid A, and pig major acute phase protein (Pig-MAP) levels using sandwich ELISA.

**Results:**

C-reactive protein and Pig-MAP were found to be significantly elevated in lame sows compared to clinically healthy sows (*p* < 0.05). The acute phase protein levels were 22.4 μg/ mL for CRP and 1.19 mg/mL for Pig-MAP in lame sows, compared to 14.7 μg/mL for CRP and 1.06 mg/mL for Pig-MAP in healthy sows.Acute phase protein levels were not associated with clinical signs of inflammation on the affected leg(s), and no differences were seen in hematology parameters between lame and healthy gestating sows.

**Conclusion:**

In this study, lameness was associated with an elevation in C-reactive protein and pig major acute phase protein.

## 1 Introduction

Lameness in gestating sows is a significant problem in intensive pig production, with studies estimating a prevalence of lameness between 6 and 23% (1–4). The variability in prevalence can be attributed to multiple factors related to reproduction stage (1), parity (5), housing (2, 6), and herd size and type (4, 7). For example, a Belgian study showed that lameness was most prevalent at entry into the gestation unit (1). Several studies have indicated loose housing (2, 6, 8) to be a risk factor for lameness, and one English study showed that larger herds and lower parity increased the risk of lameness (5). Additionally, a Danish study reported that indoor conventional production increased the risk of lameness compared to outdoor production (4). Since conventional indoor production, in large herds with group housing on slatted floors, is a very common production type in Western countries, a high prevalence of lameness in gestating sows is expected. Lameness is an economic and welfare problem and has been added to the Welfare Quality® Assessment protocols for sows (9), but it is also one of the major reasons for early culling (1, 10–12) and one of the main indicators of antibiotic use for sows in Denmark (13). The etiology of lameness is multifactorial and can be caused by infectious arthritis, osteomyelitis, trauma, osteochondrosis, osteoporosis, etc. (14). Currently, no diagnostic test exists to distinguish between bacterial and non-bacterial causes of lameness, apart from autopsy or extraction of synovial fluids from affected joints. The diagnosis is, therefore, most often based on clinical signs (14). Using clinical signs to decide on the antibiotic treatment of lameness can result in unnecessary use of antibiotics, which increases the risk of developing antimicrobial resistance (15–17). Having a non-invasive and rapid diagnostic tool that can distinguish between bacterial and non-bacterial causes of lameness, could result in reduced antibiotic use and improved sow welfare, as decisions regarding treatment or euthanization could be performed at an earlier and more informed basis. In humans and horses, studies have shown that acute phase protein (APP) levels can reflect underlying etiology with C-reactive protein (CRP) (18, 19) and serum amyloid A (SAA) (18, 20), providing a higher response in bacterial than non-bacterial cases. Furthermore, APP levels have been used to make antibiotic treatment decisions and predict prognosis in humans and horses (21). One study has investigated the correlation between lameness and APP levels in gestating sows (6). The study correlated lameness and severe lameness with CRP and haptoglobin levels, but not SAA and pig major acute phase protein (Pig-MAP), which are two other important APPs in pigs. Furthermore, the study did not correlate clinical findings of inflammation with APP levels. The primary aim of this study was to establish whether the levels of four major APPs—SAA, CRP, haptoglobin, and Pig-MAP—in pigs are elevated in lame gestating sows compared to clinically healthy gestating sows. The secondary aim was to determine differences in hematology between lame gestating sows and clinically healthy gestating sows and to correlate clinical signs of inflammation with APP levels.

## 2 Methods

### 2.1 Inclusion and herd information

A case–control study was conducted in 12 conventional sow herds in Zealand in Denmark from May 2023 to June 2023. The herds were identified with the help of a veterinary swine practitioner and selected to represent conventional Danish indoor sow herds using DanBred genetics. The herds included both those that raised their own gilts and those that purchased them. At the sow level, inclusion criteria required the sow to be in the gestation unit on the day of investigation, and that the gestation be confirmed by ultrasound. Additionally, the sow had to be either clinically healthy or lame, but without any other clinical signs of disease. Sows were excluded if they had received treatment with NSAIDS and/or antibiotics while in the gestation unit. Pens where the sows had just been placed were also excluded prior to randomization. Pens containing sows less than 3 weeks into gestation were excluded to avoid elevated acute phase protein levels caused by stress from recent mixing, which is common in group housing right after insertion into the gestation unit. Furthermore, pens with sows closer than 3 weeks to farrowing were also excluded to avoid the elevation in APP levels associated with parturition and vaccination. Information regarding herd sizes, housing type, average parity, and feeding systems are listed in Table 1.

**Table 1:** Sow and herd information including the size of the herds and the mean parity and gestation week.

Herd information		
Number of herds	12	
Size of the herds	400–1,400 year-sows	
Housing system	Group housing (20–400 sows per pen)	
Feeding system	Electronic sow feeding or floor feeding	
Sow information	Healthy	Lame
Parity	2.5	2.2
Gestation week	9.0	9.5

### 2.2 Sample size

The sample size calculation was performed for the primary aim, which was to compare acute phase protein levels in healthy and lame sows, using a comparison of sample means (22). No study was found providing mean and standard deviation (SD) of the relevant acute phase proteins for lame and healthy sows. Therefore, the mean and standard deviation (SD) of the acute phase protein levels for healthy and sick sows were estimated based on the literature investigating the APP response in healthy pigs and sows (23–30), and pigs and sows with different inflammatory conditions (23, 29, 31–46). The sample size calculation was performed with a significance level of 0.05 and a power of 80%. Due to low variation and high difference between healthy and diseased animals, the sample size required to determine CRP and SAA was 1 sow per group, which is not feasible to determine the distribution of APP levels. For haptoglobin and Pig-MAP, the difference in means was smaller, so a sample size of 49 and 51 per group was required, respectively. Additionally, other studies determining APP levels in sows were consulted (6, 23, 29, 32), and it was decided to include a total of 50 lame (cases) and 50 healthy (controls) sows from 10 different herds. Equal representation of cases and controls from each herd was ensured, as herd has a significant effect on APP levels. Due to a low number of sows fulfilling the inclusion criteria in some herds, two additional herds were included during the study.

### 2.3 Data sampling

Data were sampled by the same person in all herds. In the morning, pens containing approximately 200 gestating sows were chosen using simple randomization. After the pens were chosen, a coin flip was performed to determine whether equal or non-qual ID numbers were to be assessed. Then, the assessor (first author) entered the pens to perform the initial screening for healthy and lame sows. The initial screening focused on gait, signs of inflammation, e.g., wounds, ulcers, and lumps, and signs of illness such as diarrhea or coughing. If no clinical signs of disease were found or only lameness was detected, the ID number was noted on a sheet. When all sows had been assessed, parity, expected farrowing date, and medicine use were extracted from the herd records. Sows treated with antibiotics or NSAIDs in the gestation unit were excluded. If more than 5 healthy (control) and 5 lame (case) sows were found, during the initial screening, simple randomization was used to determine which five case sows were to be included. Matching with control sows was then performed according to parity (1, 2–3, and 4+), gestation week, and pen. Gestation week was calculated from the expected farrowing day and the assumption of a gestation length of 116 days. If less than 5 control or case sows were found, all sows in that category were included, and matching was performed as described above. After the selection process, a thorough clinical assessment of case and control sows was performed. The clinical evaluation report can be found in its full extent in Supplementary material 1. In short, the clinical assessment consisted of two parts; the first was a general clinical examination, and the second was a musculoskeletal examination conducted for only case sows. The general examination focused on behavior (activity level, orientation skills, and interest in surroundings), posture and movement (head position, claw injuries and inflammation, ability to stand and walk, and equal presentation of hind and front legs), well-being (body condition, rectal temperature, and skin color including vulva), signs of respiratory, reproductive or gastro-intestinal disease (coughing, sneezing, discharge, diarrhea, prolapse, vulva bites, or chronic mastitis), and signs of inflammation on the body (inflamed wounds, ulcers, and swellings). The musculoskeletal examination focused on lameness (degree of lameness and affected limbs) and signs of inflammation in affected limbs (swelling, redness, and wounds). The degree of lameness was described as mildly lame, where a limp was visible but the sow appeared unaffected and exercised normally. Moderate to very lame was where the sow ranged from having an obvious limp affecting the ability to exercise normally, to sows being hesitant to put weight on one or more legs and walk in a restrained manner. Severely lame sows were defined as those that were non-weight bearing on one or more legs. For control and case sows, blood was sampled from vena jugularis using an 18 G needle (BD, Mississauga, Canada), while the sow was fixated using a snout snare. Two tubes of blood were collected from each sow in one K_3_EDTA vacutainer (BD, Mississauga, Canada) for hematology analysis and one serum vacutainer (BD, Mississauga, Canada) for the acute phase protein ELISAs.

### 2.4 Hematology analysis

The hematology analysis was carried out at the Veterinary Diagnostic Laboratory, University of Copenhagen. Before the analysis, the samples were homogenously mixed using a Stuart SB3 rotator for 10 min. The laboratory analyses were performed within 24 h of sampling using an ADVIA 2120i analyzer (Siemens Healthcare A/S, Ballerup, Denmark) with multispecies software.

### 2.5 Acute phase protein analysis

The acute phase protein analysis was performed at the Technical University of Denmark. Haptoglobin concentrations were determined using a sandwich ELISA. The coating layer was an in-house mouse anti-porcine Hp monoclonal antibody and the detection antibody biotinylated commercial rabbit anti-human haptoglobin (DAKO A0030, DAKO Aps, Glostrup, Denmark) as described in Sorensen et al. (39). The assay had a detection limit of 0.13 mg/mL. A commercially available sandwich ELISA (Phase SAA assay, Tridelta Development Ltd., Kildare, Ireland) was used for the determination of SAA. The assay was originally described by McDonald et al. (47). The detection limit of the assay was 31.3 μg/mL (porcine SAA equivalents), and the samples were tested according to the manufacturer’s instructions, with the exception that the lowest dilution used was 1:20. A commercially available sandwich ELISA (Acuvet ELISA pig-MAP, Acuvet Biotech, Zaragoza, Spain) was used for the determination of Pig-MAP. The samples were tested according to the manufacturer’s instructions, with the exception that the lowest dilution used was 1/1000, resulting in a lower limit of quantification of 0.05 mg/mL. Finally, C-reactive protein concentrations were determined using a sandwich ELISA as described in Heegaard et al. (48). The assay used dendrimer-coupled cytidine diphosphocholine, polyclonal rabbit anti-human antibodies with cross-reactivity toward porcine CRP (49) and peroxidase-conjugated goat anti-rabbit antibody (DAKO Aps, Glostrup, Denmark). The standard was pooled pig serum calibrated against a human CRP calibrator (DAKO A0073, DAKO Aps, Glostrup, Denmark). The ELISA had a detection limit of 0.35 μg/mL (human equivalents). The development of plates was performed with a tetramethylbenzidine (TMB) peroxide color substrate (Kem-En-Tec Nordic Aps, Taastrup, Denmark), following the manufacturer’s instructions. The plates were read using an automatic plate reader (Thermo Multiskan Ex spectrophotometer, Thermo Scientific, Waltham, MA, USA) and values were calculated from the curve fitted to the readings of the standard (using Ascent software v. 2.6, Thermo Scientific). All samples including standards were determined in duplicate.

### 2.6 Statistical analysis

The statistical analyses were performed using R version 4.3.1 with the sows as the experimental unit. The primary objective was to determine the differences in APPs between lame and healthy sows using CRP, Pig-MAP, haptoglobin, and SAA as the response variables. This was analyzed using linear regression with group and herd and their interaction as fixed effects. To calculate the means, acute phase protein samples below the limit of detection (LOD) of the ELISA assays were given a value of 0. For the hematology analysis, a linear mixed-effects model in R was used, with group, herd, and parity as fixed effects, and pen and gestation week as random effects. The assumption of homogeneity and normality was checked using histograms, q-q plots, and residual plots. The procedure was the same for all outcomes. If normality was not fulfilled, which was the case for several of the acute phase proteins and the hematology parameters, data was log 10 transformed. ANOVA was used for model reduction, and systematic effects were excluded if not significant (*p* > 0.05), except for group, as this was the primary variable of interest. Means were extracted with EMMEANS and presented as least square means ± SEM, in the case of transformation, the results were presented as back-transformed means.

## 3 Results

### 3.1 Herds and sows

The study included 50 lame and 50 healthy sows from 12 conventional herds in Zealand, Denmark. Due to a low number of healthy sows fulfilling the inclusion criteria in the 10 original herds, it was decided to include 2 additional herds to obtain 50 healthy and 50 lame sows. From each of the 12 herds, 2 to 5 pairs of sows were included, dependent on the availability of sows that fit the inclusion criteria. Blood samples were collected from all 100 sows, except two where an EDTA sample could not be collected. Additionally, two EDTA samples were coagulated before arrival at the laboratory. Thus, 96 samples were available for the hematology analysis and 100 samples for the APP analysis. No statistically significant differences between case and control sows were found for parity (*p* = 0.1881) and gestation week (*p* = 0.3475). Descriptive data on the sows and herds is presented in Table 1.

### 3.2 Clinical signs

The included healthy sows were free from all clinical signs of disease and the lame sows were free from clinical signs of disease except for lameness. The degree of lameness was evaluated during the musculoskeletal evaluation, and the sows were categorized as mildly lame = 3, moderate to very lame (*n* = 44), and severely lame (*n* = 3). Due to the low number of mildly and severely lame sows, no conclusions on acute phase protein level and degree of lameness could be inferred from the dataset. The signs of inflammation on the limbs affected by lameness were assessed and included as signs of swelling, wounds, and redness. In total, 34 sows had no signs of inflammation on affected limbs, whereas 16 sows did.

### 3.3 Acute phase protein levels

The levels of the four major acute phase proteins in the serum from 50 clinically healthy and 50 lame sows were tested, and the means are listed in Table 2. For CRP and Pig-MAP, all 100 samples were above the LOD. For haptoglobin and SAA, 85 and 32 samples, respectively, were above the LOD. The distribution of samples above and below the LOD was identical between groups for SAA, while 41 samples from healthy sows and 44 samples from lame sows were above the LOD for haptoglobin.

**Table 2:** C-reactive protein (CRP), pig major acute phase protein (Pig-MAP), Serum amyloid A (SAA), and haptoglobin levels in healthy (*n* = 50) and lame (*n* = 50) sows.

	Healthy	Lame	SEM	*p*-value
CRP (μg/mL)^1^	14.7^a^	22.4^b^	2.11	0.0367
Pig-MAP (mg/mL)^1^	1.06^a^	1.19^b^	0.047	0.0499
SAA (μg/mL)^1^	1.61^a^	1.86^a^	0.241	0.4983
Haptoglobin (mg/mL)^1^	1.73^a^	1.86^a^	0.147	0.5433

1 The least square means have been back-transformed (logarithmic transformation). Values within a row with different superscripts differ significantly (*p* < 0.05).

A total of 28 out of 100 sows were above the 75th percentile for two or more acute phase proteins and 6 sows were above the 75th percentile for all four acute phase proteins. When comparing hematology and acute phase proteins and looking at the individual hematology profiles, no specific pattern could be detected between sows above the 75th percentile and sows below the 75th percentile for two or more APPs.

#### 3.3.1 C-reactive protein and pig major acute phase protein

**Figure 1:**
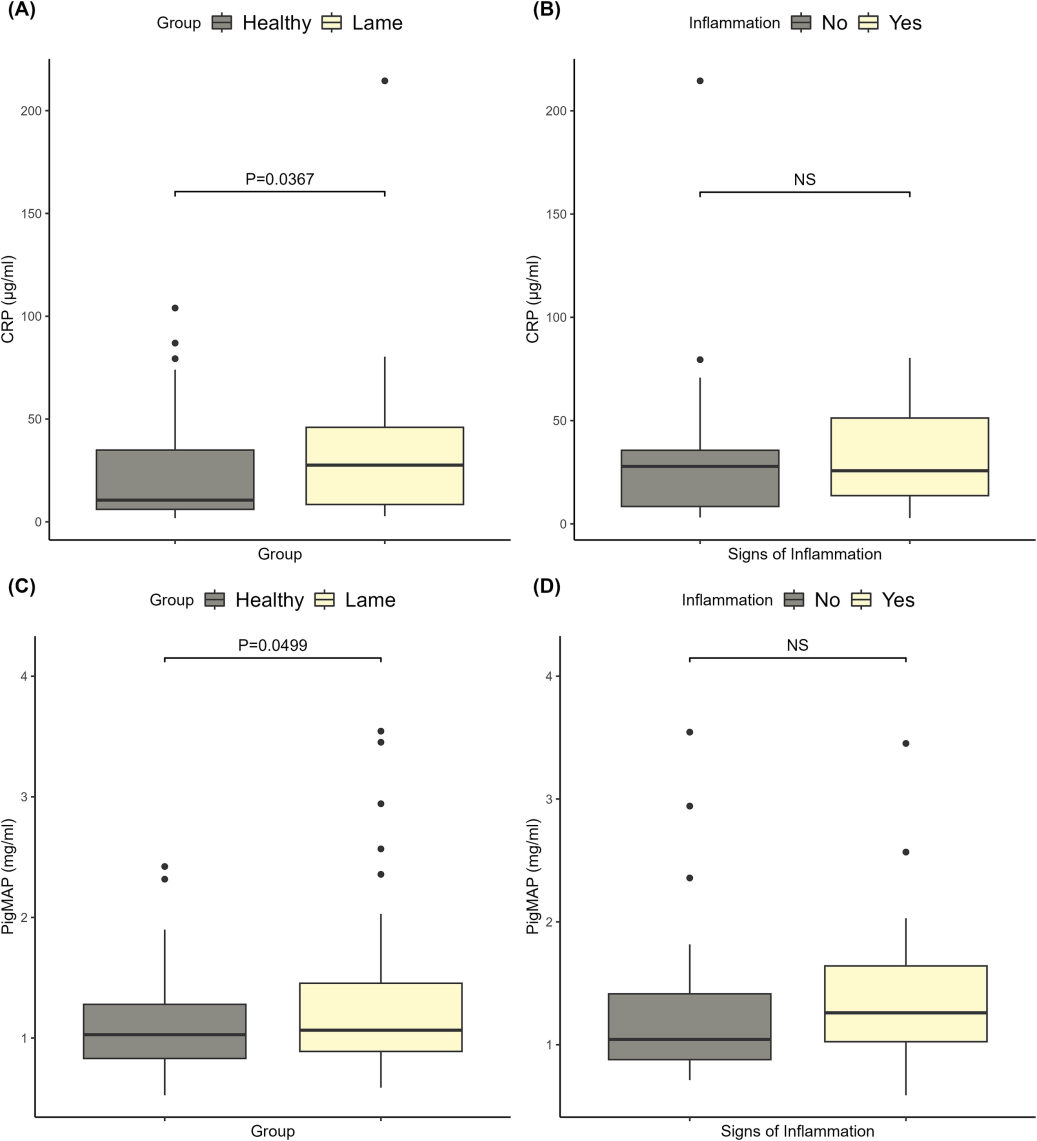
Plots of the C-reactive protein (CRP) and pig major acute phase protein (Pig-MAP) levels. **(A)** The CRP levels in healthy and lame sows, and the corresponding significance level for the difference between the two groups. **(B)** The CRP levels of lame sows with and without signs of inflammation (redness, swelling, and wounds) on the affected limbs and the corresponding significance level. **(C)** The Pig-MAP levels in healthy and lame sows, and the corresponding significance level for the difference between the two groups. **(D)** The Pig-MAP levels of lame sows with and without signs of inflammation (redness, swelling, and wounds) on the affected limbs and the corresponding significance level.

The CRP levels ranged from 1.80 to 214.50 μg/mL, and the Pig-MAP levels ranged from 0.52 to 3.54 mg/mL. Clinically healthy sows had significantly lower CRP and Pig-MAP levels than lame sows (*p* < 0.05). As can be seen from the data shown in Figures 1A,C, the two groups overlap and a clear distinction between lame and healthy sows cannot be identified based on CRP or Pig-MAP levels. Furthermore, the data in Figures 1B,D show that the signs of inflammation (swelling, redness, or wounds) on the affected limbs of lame sows were not associated with higher CRP or Pig-MAP levels.

#### 3.3.2 Serum amyloid A and haptoglobin

**Figure 2:**
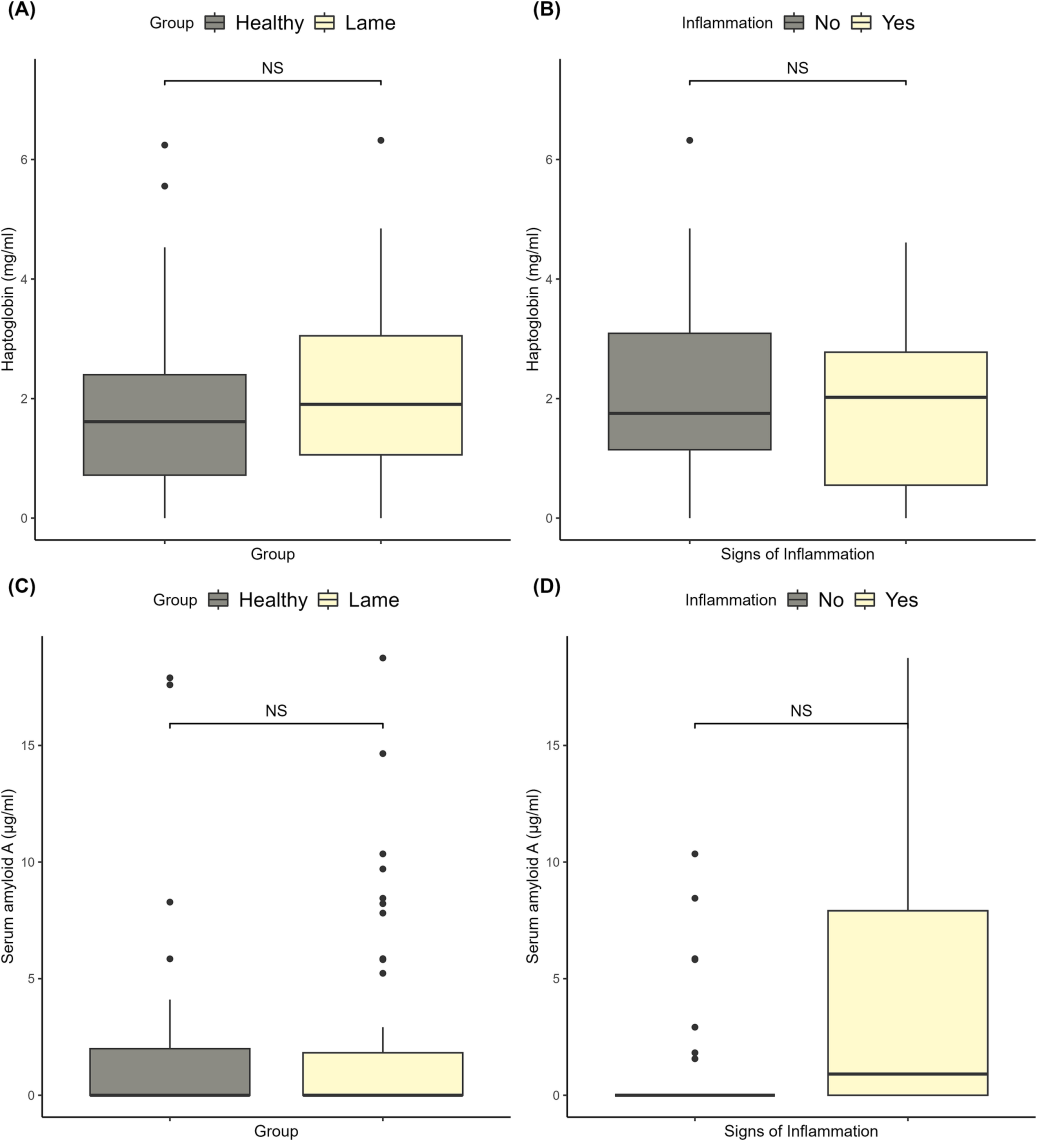
Plots of the haptoglobin and serum amyloid A levels. **(A)** The haptoglobin levels in healthy and lame sows, and the corresponding significance level for the difference between the two groups. **(B)** The haptoglobin levels of lame sows with and without signs of inflammation (redness, swelling, and wounds) on the affected limbs and the corresponding significance level. **(C)** The serum amyloid A levels in healthy and lame sows, and the corresponding significance level for the difference between the two groups. **(D)** The serum amyloid A levels of lame sows with and without signs of inflammation (redness, swelling, and wounds) on the affected limbs and the corresponding significance level.

The haptoglobin levels ranged from 0 to 6.32 mg/mL, and the SAA levels ranged from 0 to 411 μg/mL. There were no significant differences between healthy and lame sows for haptoglobin or SAA (*p* > 0.05). The levels and significance are shown in Figures 2A,C. For SAA, the outlier of 411 μg/mL was removed from the plots, as including it would make the boxplots unreadable. The outlier was included in the statistical analysis. As can be seen from the data illustrated in Figures 2B,D, the signs of inflammation had no significant effect on the haptoglobin or SAA levels.

### 3.4 Hematology analysis

A hematology analysis was performed on 21 different parameters related to erythrocytes, leukocytes, iron content, and infection status of the sows. As presented in Table 3, no statistically significant differences were found between case and control sows (*p* > 0.05), and the groups were almost indistinguishable. The mean values were comparable to the reference interval for healthy sows in mid-gestation (50), apart from the mean corpuscular hemoglobin concentration (mmol//L), which was high. The analysis revealed no indications of infections or iron deficiency in the sows.

**Table 3:** The hematology results of healthy and lame gestating sows.

	Healthy	Lame	SEM	*p*-value
*N*	46	46		
Total leukocytes, bill/L	13.8^a^	13.7^a^	0.67	0.308
Total erythrocytes, bill/L	5.41^a^	5.45^a^	0.13	0.298
Hemoglobin, mmol/L	6.92^a^	7.03^a^	0.84	0.407
Hematocrit, L/L	0.33^a^	0.33^a^	0.004	0.301
MCV, fL	60.3^a^	60.2^a^	0.77	0.430
MCHC, mmol/L	**21.1** ^**a**^	**21.2** ^**a**^	0.07	0.192
Thrombocytes, g/L	237^a^	239^a^	12.4	0.934
MPV, fL	10.4^a^	10.2^a^	0.17	0.487
MPC, g/L	248^a^	250^a^	1.41	0.234
Neutrophils, pct	40.8^a^	42.6^a^	1.37	0.587
Lymphocytes, pct	45.6^a^	44.2^a^	1.58	0.551
Monocytes, pct	3.46^a^	3.38^a^	0.173	0.744
Eosinophils^1^, pct	8.08^a^	7.54^a^	1.067	0.410
Basophils^1^, pct	0.58^a^	0.58^a^	1.06	0.679
LUC^1^, pct	0.61^a^	0.56^a^	1.129	0.675
Neutrophils^1^, bill/L	5.21^a^	5.42^a^	1.080	0.103
Lymphocytes, bill/L	5.63^a^	5.52^a^	0.510	0.652
Monocytes, bill/L	0.44^a^	0.40^a^	0.056	0.846
Eosinophils^1^, bill/L	1.14^a^	1.11^a^	1.076	0.754
Basophils^1^, bill/L	0.07^a^	0.07^a^	1.117	0.433
LUC, bill/L	0.09^a^	0.08^a^	0.014	0.849

1 The least square means have been back-transformed (logarithmic transformation). Values within a row with different superscripts differ significantly (*p* < 0.05).

Values in bold are outside the reference intervals for sows (50).

MCV, mean corpuscular volume; MCHC, mean corpuscular hemoglobin concentration; MPV, mean platelet volume; MPC, mean platelet count; LUC, large unstained cells.

## 4 Discussion

### 4.1 Results and implications for the use of APPs

In this study, we investigated the APP levels of the four moderate to major APPs for pigs. Studies on growing and finishing pigs, as well as sows, suggest that haptoglobin levels are affected by lameness (6, 34, 51). In this study, we did not find a significant difference, although a numerical difference was seen. This discrepancy could be due to the limited amount of severely lame sows, as Heinonen et al. (6) found that only severely lame sows had higher haptoglobin levels compared to healthy sows. Another explanation could be the heterogeneity within the group, as the herds varied in size, flooring type (e.g., deep bedding or concrete floor), group sizes, and feeding systems. While stress (51, 52) can affect APP levels, no studies have identified the effect of different housing types or feeding systems on APP levels. To the authors’ knowledge, SAA and its relationship with lameness in pigs have not been previously investigated. Therefore, SAA may not be affected by lameness in pigs, as opposed to what is seen in horses and cows (21). The increased CRP levels are in line with the study on sows conducted by Heinonen et al. (6), but Sanchez et al. (51) did not find significant differences in CRP levels in the saliva of lame finishing pigs. However, it should be noted that CRP levels in saliva are generally lower than those found in serum (53). While Pig-MAP has not been as extensively investigated as CRP and haptoglobin in pigs, one study did correlate Pig-MAP levels in saliva and serum with inflammatory conditions such as lameness (54) The study found that serum and salivary Pig-MAP levels were not fully correlated (*r* = 0.72). However, the raw data from experiment 1 supports our finding of a significant difference in Pig-MAP levels between healthy and lame pigs (54), although the lame sows in our study had much lower Pig-MAP levels. We also correlated the signs of inflammation on affected limbs with acute phase protein levels and found no significant differences. This could be due to a small sample size, as only 16 out of 50 lame sows had signs of redness, wounds, or swelling on the affected limb(s). It could be interesting to conduct a larger study to investigate the correlation between clinical signs of inflammation and acute phase proteins, as CRP, Pig-MAP, and SAA levels were numerically higher in the lame group with signs of inflammation compared to lame sows without signs of inflammation.

We also looked at 21 different hematologic parameters related to erythrocytes and leukocytes as well as the iron status of the sows. Comparisons were made between sows that were clinically diagnosed as either lame or healthy. No statistical difference was found between the two groups. We aimed to include sows that were similar in all aspects except for lameness, so no differences were expected in parameters related to red blood cells and iron status. However, the lack of a difference in hematologic parameters related to inflammation (e.g., total leukocytes, neutrophils, and monocytes) was unexpected. Upon investigation, we found that even within the sows where APP levels suggested an inflammatory response (e.g., the 28% where more than two APPs were in the highest 75-percentile), no clear pattern was seen in the hematologic parameters compared to sows with lower levels of APP. During the inflammatory process, a complex system of pathways is triggered (55). These will lead to increased monocytes and neutrophils in the blood and affected tissues during the acute phase of the inflammation (56). Acute lameness, independent of its etiology, should cause an inflammatory reaction, resulting in higher levels of leukocytes. Furthermore, we would believe that a proportion of the lameness would be infectious arthritis, as this is a common finding in sows euthanized because of lameness (11, 57). Infections cause an inflammatory response, therefore circulatory monocytes and neutrophils ought to increase (55). The majority of the sows included were moderately to severely lame. Only 3 out of 50 sows were non-weight bearing, indicating that the cases included in this study were not necessarily comparable to sows that are euthanized because of lameness. This could mean that the proportion of sows with infectious arthritis was lower in our study. Studies showed that non-infectious causes of lameness, such as osteochondrosis and arthroses, were reported as secondary pathological–anatomical or incidental findings in 63–90% of sows euthanized or found spontaneously dead (10, 58). Therefore, non-infectious causes appear to be common and may explain the lack of elevation in leukocytes in our study. Another factor could be that the arthritic cases were chronic as found in 34 out of 35 arthritis cases in the study by Engblom et al. (11). Chronic infections are described as low-grade inflammation and are predominated by mononuclear leukocytes such as monocytes and lymphocytes (59). Post-mortem examinations were not performed in this study, therefore the underlying etiology was not determined. Hence, we cannot infer the distribution between acute or chronic, infectious or non-infectious causes of lameness. However, the included lame sows had not been treated for lameness in the gestation unit prior to inclusion, and alterations were seen in the major acute phase proteins such as CRP and Pig-MAP. This suggests that the cases were somewhat acute, as the half-life of the major acute phase proteins tends to be short (60), and the elevation expected within 24–48 h after stimulation (61), meaning that no elevation would be expected if all cases were chronic. Furthermore, if the cases were very acute then increases in haptoglobin were not expected, as haptoglobin is considered a moderate acute phase protein in pigs and tends to peak in concentration after 3–5 days (61), perhaps further explaining a lack of difference in haptoglobin levels in this study.

### 4.2 Limitations of this study

The purpose of this study was to determine whether the four moderate to major acute phase proteins in pigs were altered during lameness in gestating sows, using the current experimental design. However, given the large overlap between sows with and without lameness, the conclusions would have strengthened if underlying etiology had been established. Post-mortem examinations of the sows were not within the scope of this study. Although the examinations could have provided an explanation for the lack of a difference in leukocyte parameters between the groups. Perhaps this also enabled us to investigate whether acute phase proteins such as CRP can be used to differentiate infectious from non-infectious causes of lameness. The study included DanBred herds that were all part of the Danish SPF system. The SPF system is a health system, with a focus on seven different pathogenic diseases (62). The included herds were free of all 7 diseases (*n* = 2), positive for one disease (*n* = 3), positive for 2 diseases (*n* = 6), and positive for three diseases (*n* = 1). The most common diseases were *Mycoplasma hyopneumoniae* and *Actinobacillus pleuropneumoniae* serotype 12 (data not shown). Studies have shown that breed (63) and health status (33) have an effect on baseline APP levels. Therefore, the acute phase protein levels obtained in this study could differ in herds with varying health statuses or genetics. To make the results more representative of herds with unknown health status or different genetics, more herds not included in the SPF system and carrying other breeds could have been included. In this study, we visited 12 conventional herds of varying sizes and production systems to ensure that the included sows were representative of conventional DanBred sows. Although 12 herds were included, only 100 samples were analyzed due to limited resources. Including more sows would have allowed us to correlate the degree of lameness with acute phase protein levels and enabled us to look more into herd differences in APP levels and hematology parameters.

## 5 Conclusion

Lame sows had elevated CRP and Pig-Map levels compared to clinically healthy sows. No differences were seen for SAA and haptoglobin levels. Signs of inflammation, such as redness, swelling, or wounds on affected limbs, were not associated with higher acute phase protein levels. Hematology parameters showed no statistically significant differences between clinically healthy and lame gestating sows.

## Data Availability

The raw data supporting the conclusions of this article will be made available by the authors, without undue reservation.

## Supplementary material

Supplementary Data Sheet 1 Clinical evaluation sheet for case and control sows.
