# Supplementary material for: Elevated levels of C-reactive protein and pig major acute phase protein in lame gestating sows
Source: Front Vet Sci. 2025 Feb 19;12:1505132. doi: 10.3389/fvets.2025.1505132 (PMC11880936; doi:10.3389/fvets.2025.1505132)
Supplement: Supplementary Data Sheet 1 — Clinical evaluation sheet for case and control sows. [file Data_Sheet_1.pdf]

Clinical exam – general

General information		Date:<br>Initials:<br>CHR:<br>Herd – ID:
Sow - Information	Sow-ID:	
	Parity:	1 ☐<br>2-3 ☐<br>4+ ☐
	Insemination date:	
	Gestation week:	
	Treated with AB in gestation unit:	
Behaviour	Does the sow show normal interest in the surroundings?<br>Yes ☐	 No ☐
	Does the sow show normal orientation skills?<br>Yes ☐	 No ☐
	Does the sow show normal activity level?<br>Yes ☐	 No ☐
Posture and movement	Normal head position?<br>Yes ☐	 No ☐
	Can the sow stand?<br>Yes ☐	 No ☐
	Equal weight on all 4 legs when standing still?<br>Yes ☐	 No ☐
	Is the sow lame?<br>Yes ☐	 No ☐
	Does the sow have claws without injuries and inflammation?<br>Yes ☐	 No ☐
	Are the hind legs and front legs equal in presentation?<br>Yes ☐	 No ☐
Body condition	Does the sow have a normal body condition?<br>Yes ☐	 No ☐*
	* If no<br>Skinny?<br>Fat?	 ☐<br>☐
Well-being	Temperature: _______________ (> 39.5˚ fever)	
	Does the sow have a normal skin colour?<br>Yes ☐	 No ☐
	Does the sow have normal respiration?<br>yes ☐	 No ☐ (e.g. shallow, forced, fast)

	Coughing?<br>Yes ☐	No ☐	
	Sneezing?<br>Yes ☐	No ☐	
	Discharge from nostrils?<br>Yes ☐	No ☐	
	Is the vulva normal in colour?<br>Yes ☐	No ☐	
GI- tract	Signs of diarrhea?<br>Yes ☐	No ☐	
	Rectal prolapse?<br>Yes ☐	No ☐	
Reproductive organs and udder	Vulva bites?<br>Yes ☐	No ☐	
	Discharge?<br>Yes ☐	No ☐	
	Yversvamp?<br>Yes ☐	No ☐	
Skin	Inflamed wounds/ulcers?<br>Yes ☐*	No ☐	
	*If yes<br>More than 1?	Yes ☐	No ☐
	Bleeding?	Yes ☐	No ☐
	> 2 cm?	Yes ☐	No ☐
	Inflamed swellings on body?<br>Yes ☐*	No ☐	
	*If yes<br>More than 1?	Yes ☐	No ☐
	Rupture/leaking?	Yes ☐	No ☐
	> 2 cm?	Yes ☐	No ☐

For case sows continue to the next section, for control sows finish here

Clinical exam – musculoskeletal system

General information		Date:<br>Initials:<br>CHR:<br>Herd – ID:
Sow - Information	Sow-ID:	

Standing position and lameness

Does the sow relieve one or more legs?

Yes ☐* No ☐

*If yes fill out table

	Midly støttehalt	Moderate to severe støttehalt	Springhalt
RF			
LF			
RH			
LH			

Inflammation

Affected leg(s) and signs of inflammation?

Yes ☐* No ☐

*if yes fill out table and circle area on illustration on next page

	RF	LF	RH	LH
Swelling				
Redness				
Wounds				
Warmth				
Pain				

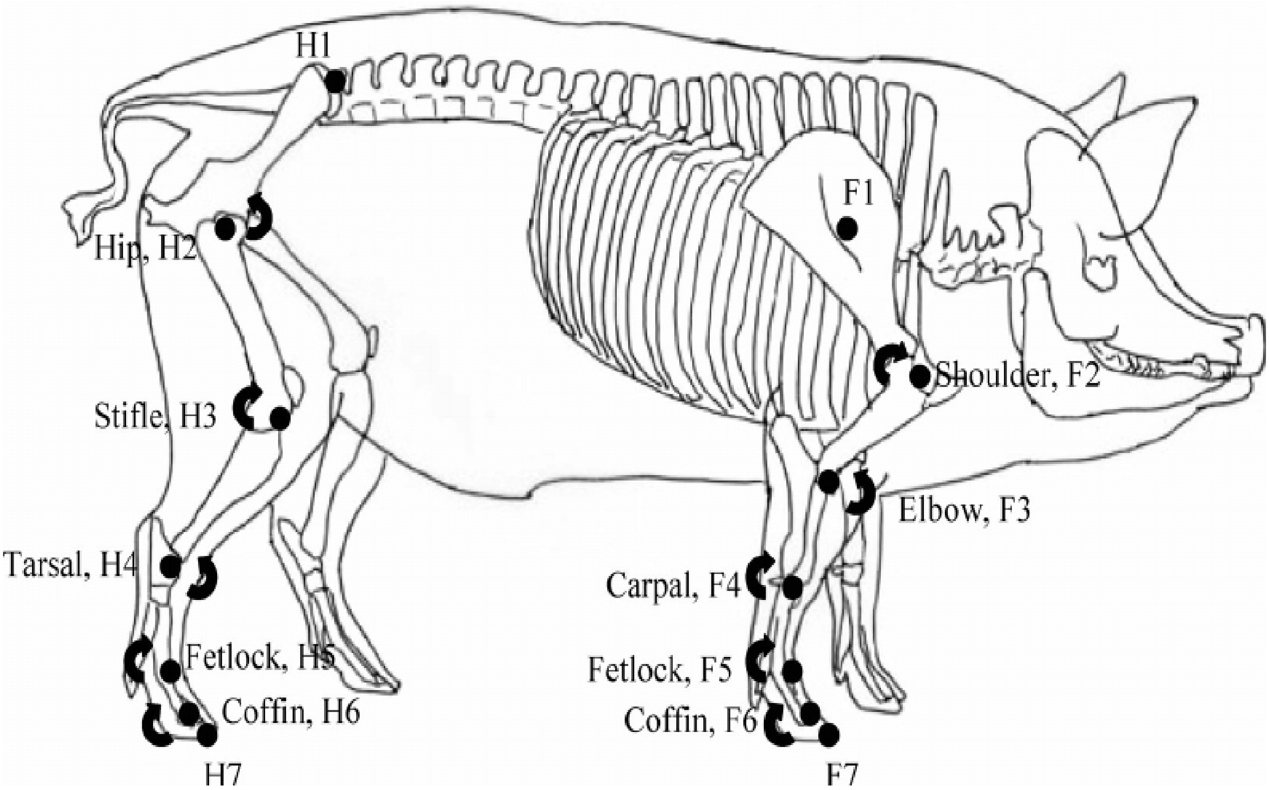

For lame sows only!

POCT	Result	Comment
Cobas b 101 CRP		
OmniChek SAA		
